# Pseudocontinuous Arterial Spin Labeling: Clinical Applications and Usefulness in Head and Neck Entities

**DOI:** 10.3390/cancers14163872

**Published:** 2022-08-11

**Authors:** Fumine Tanaka, Maki Umino, Masayuki Maeda, Ryohei Nakayama, Katsuhiro Inoue, Ryota Kogue, Makoto Obara, Hajime Sakuma

**Affiliations:** 1 Department of Radiology, Mie University School of Medicine, 2-174 Edobashi, Tsu 514-8507, Mie, Japan; 2 Department of Neuroradiology, Mie University School of Medicine, 2-174 Edobashi, Tsu 514-8507, Mie, Japan; 3 Department of Electronic and Computer Engineering, Ritsumeikan University, 1-1-1 Noji-higashi, Kusatsu 525-8577, Shiga, Japan; 4 Department of Radiology, Mie University Hospital, 2-174 Edobashi, Tsu 514-8507, Mie, Japan; 5 MR Clinical Science, Philips Japan, 2-13-37 Kohnan, Minato 108-8507, Tokyo, Japan

**Keywords:** arterial spin labeling, 3D pseudocontinuous arterial spin labeling, head and neck entity

## Abstract

**Simple Summary:**

Conventional imaging methods, such as ultrasonography, computed tomography, and magnetic resonance imaging may be inadequate to accurately diagnose lesions of the head and neck because they vary widely. Recently, the arterial spin labeling technique, especially pseudocontinuous arterial spin labeling (pCASL) with the three-dimensional (3D) readout method, has been dramatically developed to improve diagnostic performance for lesion differentiation, which can show prominent blood flow characteristics. Here, we demonstrate the clinical usefulness of 3D pCASL for diagnosing various entities, including inflammatory lesions, hypervascular lesions, and neoplasms in the head and neck, for evaluating squamous cell carcinoma (SCC) treatment responses, and for predicting SCC prognosis.

**Abstract:**

As functional magnetic resonance imaging, arterial spin labeling (ASL) techniques have been developed to provide quantitative tissue blood flow measurements, which can improve the performance of lesion diagnosis. ASL does not require contrast agents, thus, it can be applied to a variety of patients regardless of renal impairments and contrast agent allergic reactions. The clinical implementation of head and neck lesions is limited, although, in recent years, ASL has been increasingly utilized in brain lesions. Here, we review the development of the ASL techniques, including pseudocontinuous ASL (pCASL). We compare readout methods between three-dimensional (3D) turbo spin-echo and 2D echo planar pCASL for the clinical applications of pCASL to head and neck lesions. We demonstrate the clinical usefulness of 3D pCASL for diagnosing various entities, including inflammatory lesions, hypervascular lesions, and neoplasms; for evaluating squamous cell carcinoma (SCC) treatment responses, and for predicting SCC prognosis.

## 1. Introduction

Understanding the blood flow characteristics of lesions in the head and neck, as well as morphological features, is vital for accurate pretreatment diagnosis or post-treatment follow-up because blood flow is variable according to the entities of lesions and is changeable depending on the treatment response.

Among all imaging modalities that are currently feasible in clinical fields, color Doppler ultrasonography (US) is a possible option for evaluating blood flow in lesions and has been used for assessing perfusion properties of superficial organs, such as the salivary glands. However, it may be an inappropriate tool to evaluate lesions that are located in the deep structure of the head and neck [1]. As alternative tools, computed tomography (CT) perfusion, magnetic resonance (MR) dynamic contrast-enhanced (DCE) perfusion, and dynamic susceptibility contrast (DSC) perfusion may overcome this drawback of US. However, CT perfusion also requires a higher radiation dose than standard unenhanced CT [2]. Additionally, these techniques require contrast media injection and allow only one series of scans, which can hinder the application of these methods to patients with renal impairment [3,4,5]. Gadolinium-based contrast agents (GBCA) have been reportedly associated with nephrogenic systemic fibrosis (NSF), specifically in patients with reduced renal function [6,7]. The precise pathophysiology of NSF remains unknown; however, a report supported the hypothesis that prolonged GBCA exposure in patients with renal failure can be a risk for NSF [8]. Moreover, recent studies have documented gadolinium retention in the brain, bone, and skin tissues of patients without severe renal diseases [8,9]. Therefore, avoiding the unnecessary use of GBCAs would be desirable, although the clinical meaning of gadolinium retentions remains unknown.

Recently, arterial spin labeling (ASL) techniques have been clinically applied to provide noninvasive quantitative tissue perfusion assessments without contrast administration [10]. Additionally, ASL techniques allow repetitive image acquisitions, and blood flow measured by ASL is reportedly more accurate than that by DSC magnetic resonance imaging (MRI) [10,11]. This is because alterations in blood-brain barrier permeability lead to errors in calculating hemodynamic parameters in DSC perfusion MRI if not corrected [11]. The usefulness of ASL techniques has been increasingly reported in brain lesions [11,12,13,14,15,16,17,18,19,20,21,22,23,24,25,26,27,28,29], but with relatively limited reports on head and neck lesions [30,31,32,33,34,35,36,37]. Here, we review the principle of ASL and its clinical usefulness, especially that of 3D pseudocontinuous ASL (pCASL), for evaluating head and neck entities.

## 2. Principle of ASL

**Figure 1:**
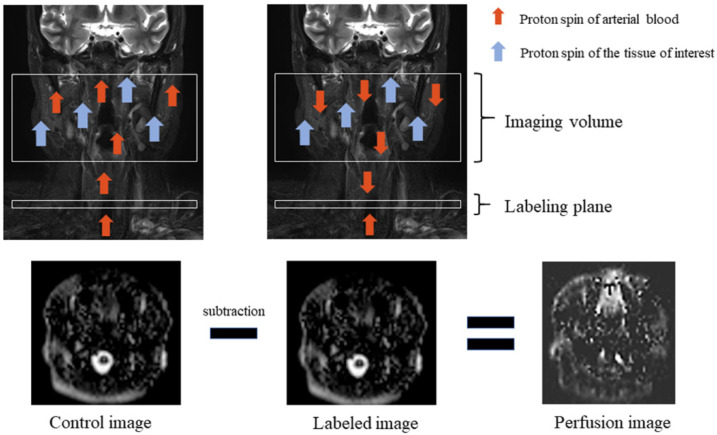
Arterial spin labeling scheme. Perfusion imaging requires control and labeled images. Proton spin of arterial blood is inverted, which is called labeling, by using radio frequency (RF) pulses in the labeling plane. Total magnetization is reduced when labeled blood travels to the tissue of interest which has its proton spin, and labeled image volume is obtained during this moment. Then, the labeled image is subtracted from the obtained control image without RF pulses, and a perfusion image is obtained.

ASL utilizes arterial blood water as an endogenous diffusible tracer by inverting blood magnetization using radio frequency (RF) pulses in the carotid and vertebral arteries [38]. Labeled images are acquired containing signals from both labeled water and static tissue water after a delay to allow for labeled blood to flow into the target tissue [38]. The signal difference between control images with and without labeling provides a measure of labeled blood from arteries delivered to the target tissue by perfusion [38] (Figure 1). In practice, the signal-to-noise ratio (SNR) of the ASL image mainly depends on underlying factors, such as the T1 relaxation time of blood and tissue, which is associated with the lifetime of the tracer and the transit time of blood that passes from the labeling plane to the imaging slice, resulting in an inevitable trade-off between the two factors [38].

ASL was initially introduced, in the 1990s, to the brain field to calculate cerebral blood flow after rat experiments [39,40], and has been developed until its application outside the brain. Firstly, ASL labeling techniques are mainly divided into three types, namely, continuous ASL (CASL), pulsed ASL (PASL), and pCASL. CASL is the first ASL method documented for perfusion quantification, which requires one single long labeling time, typically 1–3 s, using constant RF energy [38]. The signal in the image slice is recorded after the end of the labeling pulse [41]. Perfusion quantification can be falsified by magnetization transfer (MT) effects that occur due to a longer labeling pulse duration, although CASL was initially considered to be easy to implement and provide high signal gain [41,42]. Additionally, the continuous high-frequency pulse can transfer marked radiofrequency power to a patient exceeding the safety level of the specific absorption rate at higher field strengths [43].

PASL was proposed in 1994 [44,45] and employs a single short pulse with a total duration of 10–20 ms to invert a thick slab of arterial water spins, with 10–20 cm thickness [38]. The signal is recorded in the imaging slice after a defined time, which is termed the inversion time [41] or the labeling duration. There are much smaller MT effects and a lower specific absorption rate in PASL than in CASL [45]. However, the SNR of PASL is fundamentally lower than that of CASL due to shorter labeling durations and limited sizes of the labeled bolus by the spatial RF transmit coil coverage [46]. Additionally, the blood loses its label through longitudinal T1 relaxation during the inverted blood movements from the labeling region into the target tissue [47].

**Figure 2:**
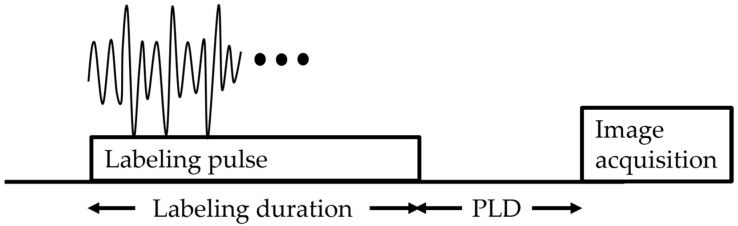
Pseudocontinuous arterial spin labeling diagram. The sequence consists of labeling, followed by a post-labeling delay (PLD), and finally image acquisition.

The pCASL technique is considered to be a combination of both CASL and PASL advantages, which employs 1000 or more shaped RF pulses at a rate of approximately 1 per ms. Thus, the temporal duration of the labeled bolus is longer in pCASL than in PASL because the blood in pCASL is continuously inverted as it flows through a labeling plane, which implies superior labeling efficiency to CASL, and causes better SNR than PASL and CASL [38,47,48]. Therefore, Alsop et al. recommended pCASL as the workhorse labeling approach for collecting ASL images [38] (Figure 2).

## 3. Timeline of the Clinical Use of ASL in the Head and Neck

The application of ASL techniques in the head and neck region was delayed although its utility was initially developed in the cerebral perfusion quantification, mainly because of less perfusion and more complex vessel anatomy in the head and neck than those in the brain, which cause lower primary signal due to low proton density and susceptibility effects [41]. Here, we review the advancements and clinical application of ASL in the head and neck, which have been previously reported based on ASL types, lesion location, and histopathological types.

To the best of our knowledge, the thyroid was the first organ in the head and neck that was documented in the clinical usefulness of ASL images [30]. In 2009, Schraml et al. reported that a quantitative PASL image of the thyroid was useful to assess autoimmune thyroid disease [30]. In 2014, Fujima et al. reported the usefulness of PASL with gradient echo (GE) and echo-planar imaging (EPI), which was considered to be a 2D readout method, for evaluating head and neck tumor viability before and after treatment [10]. Their study included patients with squamous cell carcinoma (SCC), poorly differentiated carcinoma, or adenoid cystic carcinoma, and the tumors were situated at the maxillary sinus, tongue, or oropharyngeal region [10]. They measured tumor blood flow (TBF) using a small matrix to minimize the artifacts, considering that EPI was sensitive to susceptibility artifacts by air space in the head and neck [10]. They successfully yielded the results that post-treatment TBF values were significantly lower than pretreatment TBF values and post-treatment TBF values were significantly higher in patients with residual tumors than in those without viable tumors [10]. Nevertheless, they hoped to apply a readout of the 3D spiral fast spin-echo (FSE) technique to evaluate head and neck tumors in further studies because of its benefit of fewer susceptibility artifacts [10].

In 2015, Fujima et al. reported that TBF measurement by pCASL with multi-shot spin-echo (SE)-EPI was feasible in patients with head and neck SCC [32]. They demonstrated a good correlation of TBF values between pCASL and dynamic contrast enhancement perfusion, suggesting the use of pCASL with sufficient reliability [32]. As with their study in 2014, they used a lower matrix, such as 64 × 64 or 80 × 80 in a 20–30 cm field of view (FOV), to gain higher SNR, which led to a lower number of EPI factors and subsequently minimized the susceptibility artifacts [32]. Moreover, they used parallel imaging of the acceleration factor 2 and a multi-shot readout for the ASL sequence, which also contributed to reduced EPI factors, resulting in fewer susceptibility artifacts [32].

In 2016, Fujima et al. successfully reported the usefulness of pCASL with multi-shot SE-EPI and the same matrix size and acceleration factor as those in the report in 2015 for assessing the treatment response in nonsurgical treatment [33]. Their study included patients with head and neck SCC and measured the TBF of each tumor during pretreatment and the early treatment period [33]. They concluded that the percentage change of TBF during the period was useful to determine local control [33].

Several studies, from 2015 to 2018, have reported on ASL techniques for evaluating parotid gland lesions. Kami et al. reported on the parotid blood flow of Sjögren’s syndrome glands, which were significantly higher than those of healthy glands using 3D turbo spin-echo (TSE) pCASL [34]. Kato et al. evaluated the TBF of parotid gland tumors and successfully differentiated Warthin’s tumors (WTs) from pleomorphic adenomas (PAs) and malignant tumors (MTs) using PASL sequence with GE-EPI readout [31]. Yamamoto et al. applied a 3D spiral FSE sequence to pCASL perfusion imaging and successfully showed that TBF was significantly higher in WTs than in PAs, with a positive correlation between TBF and microvessel density [49].

## 4. 3D TSE pCASL vs. 2D EP pCASL: Measurement of Blood Flow in Normal Parotid Glands

ASL signals obtained using the labeling methods can be imaged by various types of readout sequences. Readout methods for pCASL should be optimized to improve the SNR of pCASL to evaluate head and neck lesions. EPI provides high sensitivity, rapid volumetric data acquisition [50], and robustness against artifacts from motion [38]. Nevertheless, 2D multi-slice acquisitions generally have a major drawback, such as the slice dependence of the obtained perfusion SNR [50], which causes background suppression inefficiency [38]. ASL signal change is originally <1% of the background signal intensity [51]. Thus, background suppression optimization is critical especially when the ASL technique is applied to head and neck lesions because of the complex anatomy of this region, which includes air-containing structures that contribute to substantial magnetic field inhomogeneity.

Meanwhile, 3D readout methods provide higher SNR due to off-resonance artifact insensitivity [52] and superior background suppression compatibility [38]. Consequently, 3D readout methods have been adopted for ASL perfusion imaging [50,52]. Among the 3D readout methods for pCASL, spiral FSE imaging has been frequently used, which provides high SNR images because the largest signal through the signal sampling is in the center of the *k*-space [53]. However, the signal level in the periphery of the *k*-space is very low, which can be problematic [53]. Whereas, TSE readout obtains a high signal echo in the periphery of the *k*-space, as well as its center using an optimized-variable flip-angle scheme [53]. Several reports have described that TSE methods improved the geometrical accuracy, and therefore, could be applied in salivary gland imaging [54,55].

We previously evaluated the difference between the image quality of pCASL with 2D EP and 3D TSE using Cartesian acquisition [56]. All images were obtained using a 3.0 T MR scanner (Ingenia; Philips Medical Systems, Best, The Netherlands) with a head/neck coil. We summarize the main recommended parameters for 2D EP pCASL and 3D TSE pCASL at 3.0 T (Table 1). The labeling plane is set parallel to the imaging volume and perpendicular to the common carotid artery.

**Figure 3:**
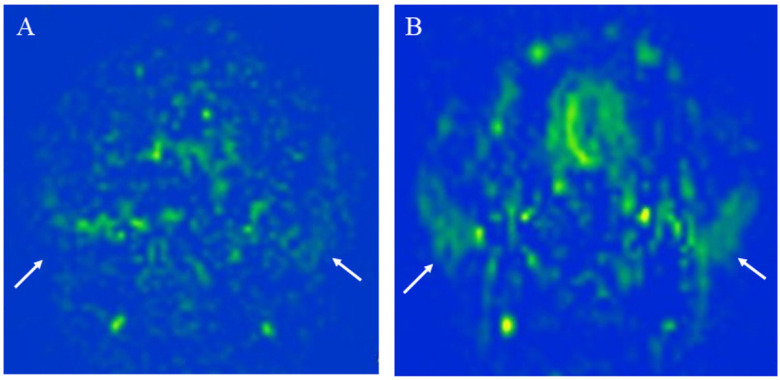
The measurement of the parotid gland blood flow of a 27-year-old healthy female using: (**A**) 2D echo planar (EP) pseudocontinuous arterial spin labeling (pCASL) image; (**B**) 3D turbo spin-echo (TSE) pCASL image. The 3D TSE pCASL image depicts bilateral normal parotid blood flow better than the 2D EP pCASL image (arrows). The parotid blood flow value on the 3D TSE pCASL image (right mean of 56.85 mL/100 g/min and left mean of 47.46 mL/100 g/min) is greater than on the 2D EP pCASL image (right mean of 43.07 mL/100 g/min and left mean of 30.39 mL/100 g/min).

TBF is calculated according to the following equation [38]:

$$\mathrm{TBF}=\frac{6000\;\cdot \lambda \cdot \left({\mathrm{SI}}_{\mathrm{control}}-{\mathrm{SI}}_{\mathrm{label}}\right)\;{\cdot e}^{\frac{\mathrm{PLD}}{T_{1,\;\mathrm{blood}}}}}{2\cdot \alpha \cdot T_{1,\;\mathrm{blood}\;}{\cdot \mathrm{SI}}_{\mathrm{PD}\;}\cdot \left(1-e^{-\frac{\tau }{T_{1,\mathrm{blood}}}}\right)}\;[\mathrm{mL}/100\;g/\min]$$

where λ is the blood/tumor-tissue water partition coefficient (1.0 g/mL), and SI_control_ and SI_label_ are the time-averaged signal intensities in the control and label images, respectively. T_1,blood_ is the longitudinal relaxation time of blood (1650 ms), α is the labeling efficiency (0.85), SI_PD_ is the signal intensity of a proton density-weighted image, and τ is the label duration (1650 ms). The value of λ was 1.0 mL/g. We used the same model and conditions as those used for calculating blood flow in the brain to calculate TBF. We show that 3D TSE pCASL has a better head and neck blood flow delineation than 2D EP pCASL with higher SNR and less image distortion (Figure 3).

## 5. Clinical Applications of 3D TSE pCASL in the Head and Neck

### 5.1. Clinical Application for Diagnosis

#### 5.1.1. Inflammatory Lesions: Sialadenitis and Dacryoadenitis

**Figure 4:**
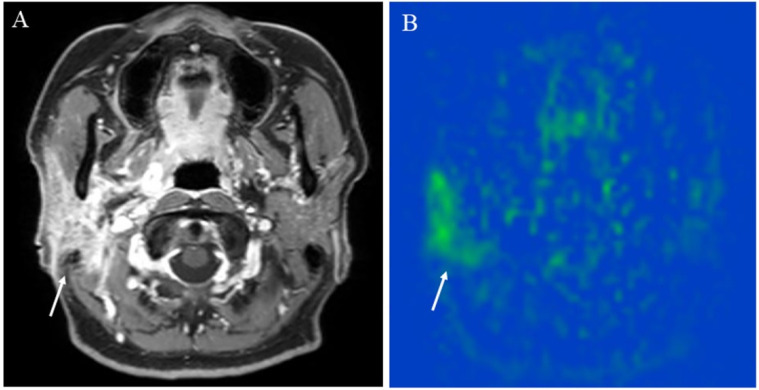
A 48-year-old female with right chronic parotitis: (**A**) Contrast-enhanced T1-weighted image shows intense right parotid gland enhancement (arrow); (**B**) pCASL image shows higher blood flow within the lesion (arrow) (right mean of 81.17 mL/100 g/min) than the opposite parotid gland (left mean of 36.82 mL/100 g/min).

Sialadenitis is a common pathology that affects the salivary glands. Acute infective parotitis shows increased vascularization with enlarged glands, which can be demonstrated using US echo with color Doppler [57]. A study reported on the usefulness of ASL for evaluating central nervous system infection [24]. Similarly, chronic parotitis has been demonstrated by pCASL (Figure 4), which was more clearly depicted than in the US because the MRI showed the whole lesion, including the superficial and deep parotid gland lobes.

**Figure 5:**
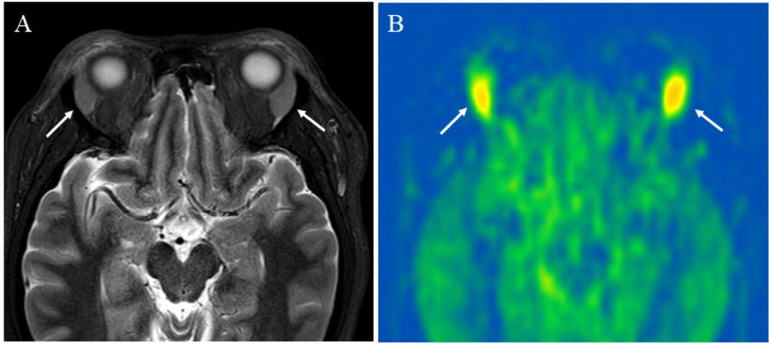
A 43-year-old female with immunoglobulin G4-related disease that involves the bilateral lacrimal gland: (**A**) Short tau inversion recovery (STIR) shows bilaterally enlarged lacrimal glands with high signal intensity (arrows); (**B**) pCASL image shows high blood flow within the lesions (arrows) (right mean of 65.70 mL/100 g/min and left mean of 66.98 mL/100 g/min).

ASL may also be useful for evaluating systemic inflammatory disease that manifests as sialadenitis. Immunoglobulin G4 (IgG4)-related disease is a systemic fibro-inflammatory disease, which can affect the salivary glands in more than 30–40% of patients, submandibular glands more often than parotid glands, and bilateral salivary glands more often than unilateral salivary gland [58]. Other than salivary gland lesions, the most commonly involved organ is reportedly the lacrimal gland [59] (Figure 5). Fluorodeoxyglucose positron emission tomography/CT (FDG-PET/CT) has been reported to evaluate not only the head and neck lesions but also all potential lesions throughout the body to diagnose and evaluate IgG4-related disease activity [60,61]. However, FDG-PET/CT requires radiation exposure, and consequently, follow-up examination with FDG-PET/CT after treatment should be limited. Another method to evaluate the lesions of IgG4-related disease is the US, which revealed the features of IgG4-related disease in the salivary glands, such as increased color Doppler signaling ratios; however, the US may be inappropriate for depicting lesions in deep locations, such as the orbital foramen [62]. The ASL image of sialadenitis has been reported on Sjögren’s syndrome by Kami et al. [34]. Schraml et al. reported the usefulness of ASL for evaluating thyroid perfusion in patients with autoimmune thyroid disorders, such as Graves disease or Hashimoto’s thyroiditis [30]. Their study showed significantly elevated perfusion values in patients with these autoimmune thyroid disorders as compared with perfusion values in healthy controls [30]. Considering these reports where perfusion image in the head and neck was successfully depicted, the ASL method can demonstrate its advantage of repeatability without radiation exposure and superiority for showing deeply located lesions for evaluating sialadenitis or dacryoadenitis over US. Nonetheless, inflammatory findings are nonspecific and must be held up against the clinical manifestation [63].

#### 5.1.2. Hypervascular Lesions

Vascular lesions are rare entities in the head and neck but can be clearly depicted by a pCASL image if the lesions have the hypervascularity feature. These lesions can affect the pediatric population; thus, noninvasive methods for diagnosis should be prioritized before endovascular treatment or surgery. Regarding brain lesions, Kishi et al. reported a case of hemangioblastoma in the cerebellopontine angle showing extremely high tumor blood flow on the pCASL image, which provided additional useful information for the differential diagnosis of schwannoma and meningioma [29]. Likewise, pCASL might be useful for differentiating hypervascular tumors in the head and neck.

**Figure 6:**
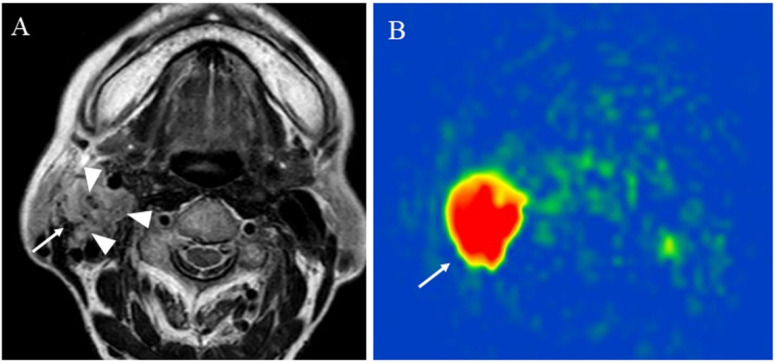
An 83-year-old female with the right carotid body paraganglioma: (**A**) T2-weighted image shows a right cervical mass (arrow) at the carotid artery bifurcation with high signal intensity and flow voids or salt and pepper appearance within the mass (arrowhead); (**B**) pCASL image shows abundant blood flow within the lesion (arrow) (mean of 303.04 mL/100 g/min).

**Figure 7:**
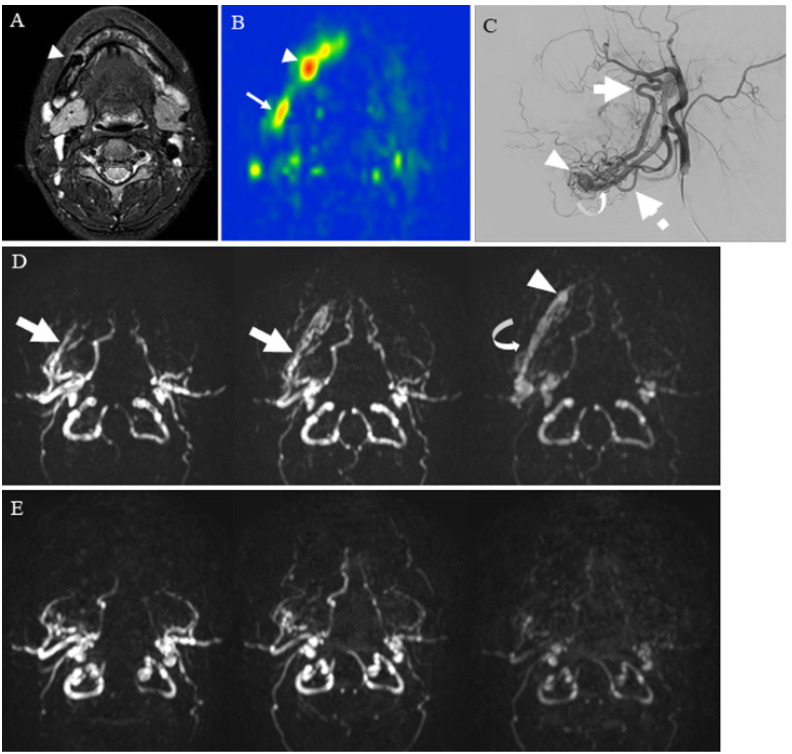
A 24-year-old female with mandibular arteriovenous malformation (AVM): (**A**) STIR shows an intraosseous flow void of the jaw (arrowhead), suggestive of AVM nidus; (**B**) pCASL image shows markedly elevated blood flow of the nidus (arrowhead) (mean of 374.83 mL/100 g/min) and high blood flow surrounding the nidus (arrow); (**C**) right external carotid angiography reveals that the AVM is predominantly supplied by the right inferior alveolar artery (thick arrow) and mental artery (dashed arrow) with drainage into the right inferior alveolar vein (curved arrow) via the nidus (arrowhead); (**D**) contrast inherent inflow-enhanced multiphase angiography (CINEMA) shows that the AVM is supplied by the right inferior alveolar artery (thick arrows) and drained into the right inferior alveolar vein (curved arrow) via the nidus (arrowhead) before treatment; (**E**) after the right alveolar artery and vein embolization, no feeder, drainage vein, or nidus is depicted.

**Figure 8:**
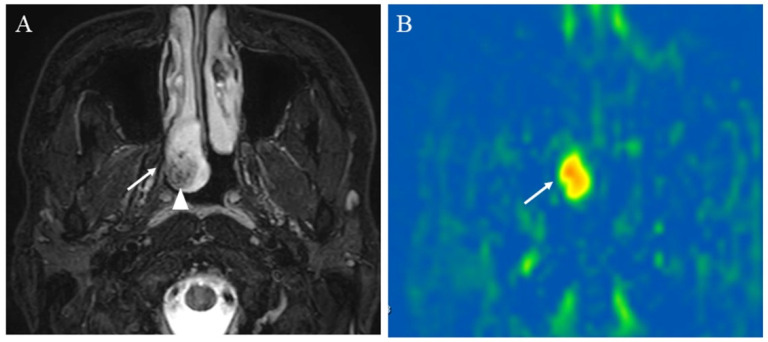
A 23-year-old male with juvenile nasopharyngeal angiofibroma: (**A**) STIR shows a mass with high signal intensity (arrow) and subtle flow voids within the mass (arrowhead); (**B**) pCASL image shows markedly elevated blood flow within the lesion (arrow) (mean of 380.42 mL/100 g/min).

Carotid body paraganglioma (Figure 6), arteriovenous malformation (AVM) (Figure 7), and juvenile nasopharyngeal angiofibroma (JNA) (Figure 8) show apparent high TBF or lesion blood flow on an ASL image. Conventional MRIs, such as T1-weighted and T2-weighted images, may show a “salt and pepper appearance” within paraganglioma [64], AVM nidus, and its draining vein [28], and intratumoral signal void within JNA [65]. However, these features may be seen in larger lesion sizes. Le et al. reported on ASL application to intracranial AVMs or dural arteriovenous fistulas (AVFs) with a range of sizes from large to small, and ASL with the 3D FSE readout method could identify venous signal intensity due to shunting vascular structure even if lesions were smaller than 2 cm [16]. Likewise, an ASL image may tremendously contribute to its diagnosis if a lesion, suspected of these vascular lesions, appears as a small head and neck mass. Additionally, ASL helps in diagnosing residual or recurrent lesions after treatment by detecting the blood flow within a lesion [28].

As an advanced ASL technique, contrast inherent inflow-enhanced multiphase angiography (CINEMA), introduced by Nakamura et al., provides the information on hemodynamics and structure of vessels in the target tissue without contrast agents [66,67]. The CINEMA technique requires PASL or pCASL tagging scheme and provides multi-timepoint data by varying the delay time interval between labeling and imaging at the slices of interest [68,69]. Iryo et al. reported excellent agreement between CINEMA and digital subtraction angiography in visualizing the site of dural arteriovenous fistulas, including the fistula, the feeding arteries, and the draining veins [70]. Hu et al. successfully documented the intracranial AVM case that showed the nidus and the feeder arteries on CINEMA [68]. This technique can be applied to head and neck AVM, as shown in Figure 6. More recently, 4D pCASL-based angiography using CENTRA-keyhole and view-sharing (4D-PACK) and vessel-selective 4D-PACK (4D-S-PACK) have been introduced to provide better intracranial peripheral artery visualization as compared with CINEMA [69,71]. Togao et al. reported the usefulness of 4D-S-PACK for assessing hemodynamics in Moyamoya disease, brain AVM, and intracranial dural AVF [72,73,74]. Whereas, 4D-PACK or 4D-S-PACK technique has not yet been applied to head and neck lesions.

#### 5.1.3. Salivary Gland Tumor Differentiation: Malignant Salivary Tumors, Pleomorphic Adenomas, and Warthin’s Tumors

Differentiating MTs from benign tumors, which comprise 45% of PAs, followed by WTs, is essential among various types of salivary gland tumors. Kato et al. successfully showed that the ASL method was useful in differentiating WTs from PAs and MTs using tumor-to-parotid gland signal intensity ratios (SIRs) to facilitate salivary tumor diagnosis [31]. However, SIRs on ASL between PAs and MTs were not significantly different [31]. This is because its qualitative analysis method and PASL sequence with the GE-EPI readout was used in the study, which had a lower SNR than that of pCASL [31]. Yamamoto et al. reported that the mean and maximum calculated TBF using pCASL images were significantly higher in WTs than in PAs [49]. Takumi et al. reported no significant difference in the mean TBF between MTs and benign salivary lesions, including nontumor lesions, such as IgG4-related disease, Kimura’s disease, and cavernous hemangioma [75]; both studies by Yamamoto et al. and Takumi et al. used the mean value of TBF, which may not sufficiently show tumor heterogeneity.

**Figure 9:**
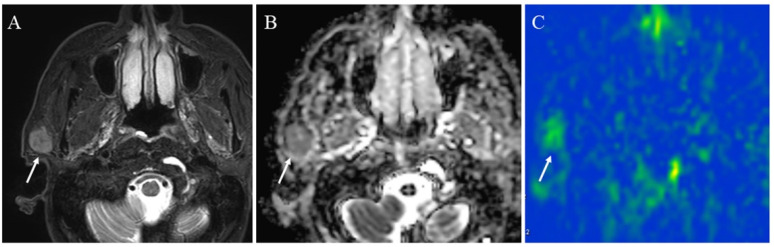
A 72-year-old male with acinic cell carcinoma of the right parotid gland: (**A**) STIR shows a mass with high signal intensity (arrow); (**B**) ADC map shows mild restricted diffusion (arrow) (ADC 10th percentile, 0.93 × 10^−3^ mm^2^/s and ADC mean of 1.03 × 10^−3^ mm^2^/s); (**C**) pCASL image shows high TBF (arrow) (TBF 50th percentile, 34.26 mL/100 g/min).

**Figure 10:**
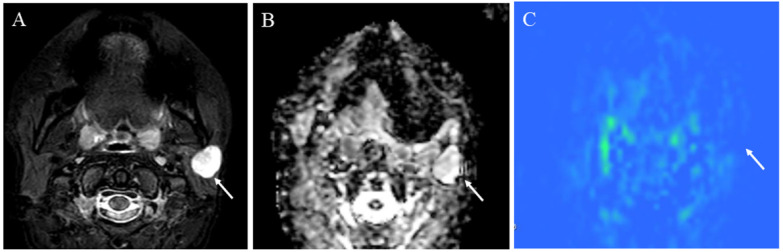
A 41-year-old female with pleomorphic adenoma of the left parotid gland: (**A**) STIR shows a mass with bright signal intensity (arrow); (**B**) ADC map shows increased diffusion (arrow) (ADC 10th percentile, 1.32 × 10^−3^ mm^2^/s and ADC mean of 1.48 × 10^−3^ mm^2^/s); (**C**) pCASL image shows low TBF (arrow) (TBF 50th percentile, 9.75 mL/100 g/min).

**Figure 11:**
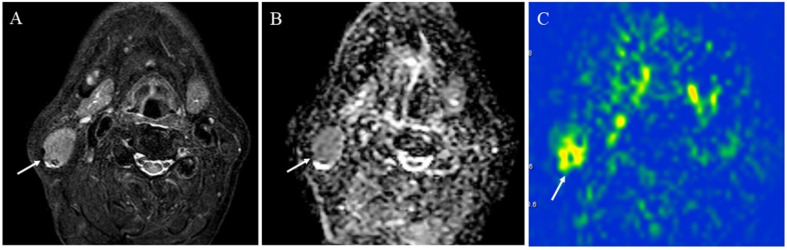
A 75-year-old male with Warthin’s tumor of the right parotid gland: (**A**) STIR shows a mass with high signal intensity (arrow); (**B**) ADC map shows restricted diffusion (arrow) (ADC 10th percentile, 0.70 × 10^−3^ mm^2^/s and ADC mean of 0.85 × 10^−3^ mm^2^/s); (**C**) pCASL image shows high TBF (arrow) (TBF 50th percentile, 150.53 mL/100 g/min).

Our previous study utilized histogram analysis for evaluating the difference of TBF measured by 3D TSE pCASL among PAs, WTs, and MTs. Histogram analyses are considered to be strong and reliable quantitative surrogate markers of tumor heterogeneity [76]. As a result, the mean, and 50th, 75th, and 90th percentiles of TBF showed significant differences among all three tumor types [37]. In our study, the 50th percentile of TBF was the most reliable parameter of all TBF histogram parameters when differentiating MTs from WTs and PAs because of receiver operating characteristic curve analysis, wherein MTs (Figure 9) showed a higher 50th percentile of TBF than PAs (Figure 10) and lower than WTs (Figure 11) [37]. The optimal cutoff values were 20.06 mL/100 g/min for MTs and PAs and 78.02 mL/100 g/min for MTs and WTs [37]. The 90th percentile of TBF when differentiating WTs from PAs was the most reliable out of all TBF parameters, and WTs showed a higher 90th percentile of TBF than PAs [37]. The optimal cutoff value was 82.30 mL/100 g/min [37]. Yamamoto et al. showed that the higher TBF value of WTs than of PAs was attributable to higher microvessel density in WTs than in PAs [49]. Thus, TBF provides useful information on the vascularity of salivary gland tumors for their differential diagnosis. Meanwhile, the apparent diffusion coefficient (ADC) map has also been reportedly more reliable than TBF when differentiating PAs from WTs because the ROC analysis of ADC_all_, which represented all parameters of ADC in combination, showed the best diagnostic performance [37]. Reportedly, it is because histopathologically, PAs comprise an abundant myxoma-like stroma, which probably contributed to a higher ADC value than WTs [31]. We obtained the results of an increased AUC value for the combination of TBF_all_, which represented all parameters of TBF combined, and ADC_all_ as compared with the AUC value for TBF_all_ or ADC_all_ alone for differentiating MTs from PAs and WTs [37]. We concluded that the combination of TBF measured by 3D TSE pCASL image and ADC map evaluated by histogram analysis would help differentiate MTs from PAs and WTs [37].

#### 5.1.4. Other Head and Neck Tumors

**Figure 12:**
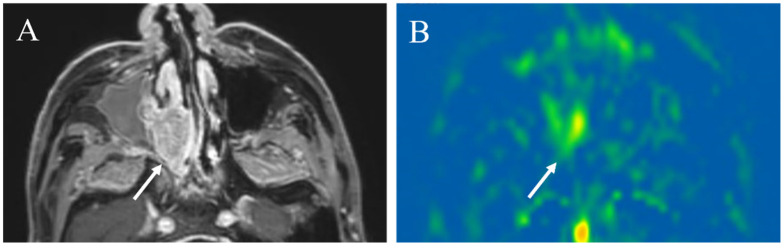
A 87-year-old male with squamous cell carcinoma in the right nasal cavity: (**A**) Contrast-enhanced T1-weighted image shows an enhanced mass (arrow); (**B**) pCASL image shows high blood flow within the lesion (arrow) (TBF mean of 70.17 mL/100 g/min) (arrow).

**Figure 13:**
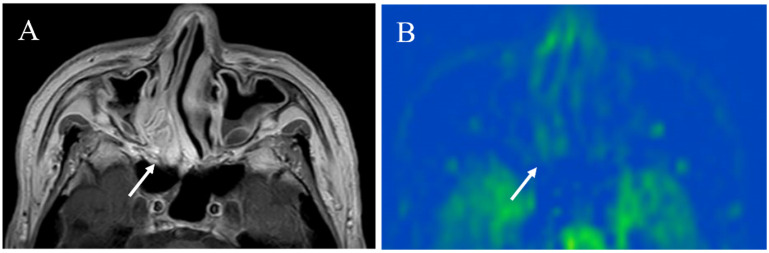
A 73-year-old male with inverted papilloma: (**A**) Contrast-enhanced T1-weighted image shows an enhanced mass with convoluted cerebriform pattern (arrow); (**B**) pCASL image shows a slight increase in blood flow within the lesion (arrow) (TBF mean of 39.46 mL/100 g/min).

pCASL has been also reported to be useful in differentiating other head and neck tumors. SCC is the most common malignant tumor, and inverted papilloma is the most common benign tumor in the nasal or sinonasal cavity [77]. Fujima et al. reported that the mean TBF derived from pCASL image was significantly higher in SCC than inverted papilloma, i.e., 141.2 ± 33.1 vs. 77.8 ± 31.5 mL/100 g/min, and pCASL could be useful to differentiate SCC from inverted papilloma [77] (Figure 12 and Figure 13). They also reported that SCC showed significantly higher mean TBF and histogram coefficient variation (CV) than malignant lymphoma in the nasal or sinonasal cavity, i.e., 140.6 ± 35.7 vs. 93.8 ± 15.1 mL/100 g/min for mean TBF, and 0.45 ± 0.09 vs. 0.33 ± 0.04 for CV [78].

Ahn et al. evaluated the histogram TBF parameters derived from pCASL between human papillomavirus (HPV)-positive and -negative in oropharyngeal SCC [79]. Oropharyngeal SCC with HPV-positive showed significantly lower standard deviation (SD) and 95th percentile of TBF, i.e., 27.8 vs. 37.7 mL/100 g/min for SD and 111.7 vs. 147.3 mL/100 g/min for 95th percentile of TBF [79].

### 5.2. Evaluation of Squamous Cell Carcinomas Treatment Response

**Figure 14:**
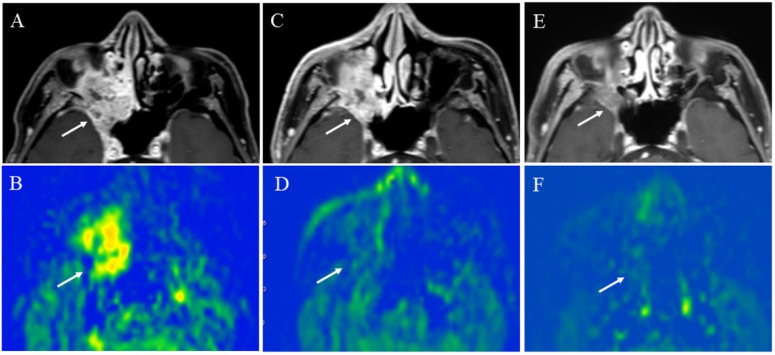
A 64-year-old male with squamous cell carcinoma of the right maxillary sinus: (**A**) Pretreatment contrast-enhanced T1-weighted image shows a poorly marginated mass (arrow); (**B**) the pCASL image shows high TBF (arrow) (mean of 102.9 mL/100 g/min). During chemoradiotherapy, the tumor slightly decreased in size (arrow) (**C**), whereas significantly decreased in TBF (arrow)(mean of 44.8 mL/100 g/min) (**D**). After chemoradiotherapy, the tumor decreased in size (arrow) (**E**) and further decreased in TBF (arrow) (mean of 16.7 mL/100 g/min) (**F**).

Fujima et al. revealed that a decreased TBF in patients with head and neck SCC reflected a treatment effect, such as a decreased intratumoral arteriovenous shunt or a reduced vascular bed volume after chemoradiotherapy or endothelial cell damage of blood vessels by radiation therapy [33]. These tumor changes were likely observed before the tumor volume reduction. Therefore, we suggest that 3D TSE pCASL may be beneficial in evaluating a decreased TBF, which may help predict tumor volume reduction afterward (Figure 14).

### 5.3. Prediction of Squamous Cell Carcinomas Prognosis

Fujima et al. applied 2D EP pCASL to measure the TBF of SCC as a prognostic indicator and reported that lower pretreatment TBF likely indicated a poor prognosis in patients with SCC in the head and neck regions, such as nasal or sinonasal cavity and oropharynx, who were treated with superselective arterial infusions of cisplatin with concomitant radiation therapy [33]. Additionally, the percentage change of TBF in the group of failure in treatment was smaller than that in the local control group [33]. Cao et al. applied 3D fast spin-echo spiral-based pCASL and showed that the pretreatment TBF of patients in the partial response group was significantly lower than that in the complete response group, i.e., 76.56 ± 26.23 vs. 89.43 ± 20.56 mL/100g/min [80]. In addition, the reduction rate of TBF between mid- or post-treatment and pretreatment was significantly lower in complete response than those in partial response, i.e., −36.49 ± 18.27% vs. −11.80 ± 20.74% [80].

Fujima et al. also showed that an increased TBF in patients with a good prognosis could be observed just after the second arterial cisplatin infusion of superselective arterial infusions of cisplatin with concomitant radiation therapy, which was consistent with the previous study with CT perfusion [33,81]. They hypothesized that the good prognosis in patients with an increased TBF was attributed to increased tumor oxygenation, which affected the treatment response sensitivity in radiation therapy [33]. They speculated that the initially increased TBF was caused by decreased tumor internal pressure, derived from the initial treatment effect of cytotoxicity by chemoradiotherapy, which resulted in decreased intratumoral artery compression [33].

More recently, machine learning (ML)-based analyses have been increasingly applied for evaluating various types of lesions. Fujima et al. revealed an ML algorithm combining MRI data including pCASL, DWI, and conventional MRI, such as T1WI and T2WI could highly predict the treatment outcome in patients with sinonasal SCCs [36]. In their study, ML was used to select the five top-ranked features in training datasets which included not only the functional parameters such as TBF, but also other parameters such as the morphological parameter [36]. ML methods can possibly improve the objective evaluation of images because the data are randomly split into training and validating, and diagnostic accuracy is evaluated using a cross-validation scheme to find the combination of features with the highest predictive power [36].

Finally, Table 2 shows the main clinical ASL papers discussed in this review.

## 6. Conclusions and Future Perspectives

ASL is an emerging technique that provides perfusion information of lesions. We successfully obtained images with excellent SNR by using pCASL with the 3D TSE readout method, although the application of this technique to the head and neck region was initially difficult as compared with the brain. Thus, we demonstrated that this method might enhance the diagnostic performance of critical lesions, such as inflammation, vascular lesions, salivary gland tumors, and head and neck SCCs. ASL can provide useful blood flow-related information in a lesion with high sensitivity, but the drawback includes intrinsically inadequate anatomical and morphological data. Thus, a machine learning-based analysis should be applied to diverse head and neck lesions in the future to obtain quantitative data from both morphological and functional MRI sources, including ASL images, which may further improve diagnostic accuracy.

## Figures and Tables

**Table 1:** pCASL imaging protocol.

	2D EP	3D TSE
Repetition time/echo time	4500/14	6000/40
Number of excitations	36	3
Dynamic scan time	0:09	1:48
EPI factor	35	100
Flip angle (°)	70	90
Matrix	80 × 80	80 × 80
Field of view (mm)	240	240
Label gap (mm)	73.36	73.36
Labeling duration (ms)	1650	1650
Post-labeling delay (ms)	1800	1800
Scan time	5:33 for 22 slices	5:36 for 44 slices

Note: pCASL, pseudocontinuous arterial spin labeling; EP, echo planar; TSE, turbo spin-echo.

**Table 2:** Table summarizing the main ASL papers regarding clinical applications to the head and neck region.

Main Findings	First Author
PASL for evaluating thyroid perfusion in autoimmune thyroid disease	Schraml, C. et al. *Radiology* 2009 [30]
PASL for evaluating H&N tumor viability before and after treatment	Fujima, N. et al. *J. Magn. Reson. Imaging* 2014 [10]
TBF in H&N SCC by pCASL as compared with DCE perfusion	Fujima, N. et al. *J. Magn. Reson. Imaging* 2015 [32]
pCASL for assessing the treatment response in H&N SCC	Fujima, N. et al. *AJNR Am. J. Neuroradiol.* 2016 [33]
pCASL for evaluating parotid gland of Sjögren’s syndrome	Kami, Y.N. et al. *PLoS ONE* 2016 [34]
PASL for differentiating WT from PA and MT in parotid gland	Kato, H. et al. *Eur. Radiol.* 2015 [31]
pCASL for differentiating H&N SCC from inverted papilloma	Fujima, N. et al. *Dentomaxillofac. Radiol.* 2015 [78]
pCASL for differentiating H&N SCC from malignant lymphoma	Fujima, N. et al. *Eur. J. Radiol.* 2015 [79]
pCASL for differentiating WT from PA, evaluating correlation between TBF and microvessel density	Yamamoto, T. et al. *Neuroradiology* 2018 [49]
Machine learning using pCASL for prediction of treatment outcome in SCC	Fujima, N. et al. *Cancers* 2019 [36]
pCASL for differentiating HPV status using histogram analysis	Ahn, Y. et al. *Neuroradiology* 2021 [80]
pCASL for assessing the treatment response in H&N SCC	Cao, X. et al. *Front. Oncol.* 2021 [81]
pCASL for differentiating MT from PA and WT using histogram analysis	Tanaka, F. et al. *Sci. Rep.* 2022 [37]

Note: PASL, pulsed arterial spin labeling; H&N, head and neck; TBF, tumor blood flow; SCC, squamous cell carcinoma; pCASL, pseudocontinuous arterial spin labeling; DCE, dynamic contrast enhancement; WT, Warthin’s tumor; PA, pleomorphic adenoma; MT, malignant tumor; HPV, human papillomavirus; J Magn Reson Imaging, Journal of Magnetic Resonance Imaging; AJNR Am J Neuroradiol, American Journal of Neuroradiology; Eur Radiol, European Radiology; Dentomaxillofac Radiol, Dentomaxillofacial Radiology; Eur J Radiol, European Journal of Radiology; Front Oncol, Frontiers in Oncology; Sci Rep, Scientific Reports.
